# Alteration of NF-κB activity leads to mitochondrial apoptosis after infection with pathological prion protein

**DOI:** 10.1111/j.1462-5822.2007.00950.x

**Published:** 2007-06-15

**Authors:** Soizic Bourteele, Katja Oesterle, Andreas O Weinzierl, Stephan Paxian, Marc Riemann, Roland M Schmid, Oliver Planz

**Affiliations:** 1 Friedrich-Loeffler-Institut, Federal Research Institute for Animals Health, Institute of Immunology Tübingen Germany; 2 Department of Immunology, Institute for Cell Biology, Eberhard-Karls-University Tübingen Germany; 3 Department of Internal Medicine II, Technical University Munich, Germany

## Abstract

Nuclear factor kappa B (NF-κB) is a key regulator of the immune response, but in almost the same manner it is involved in induction of inflammation, proliferation and regulation of apoptosis. In the central nervous system activated NF-κB plays a neuroprotective role. While in some neurodegenerative disorders the role of NF-κB is well characterized, there is poor knowledge on the role of NF-κB in prion disease. We found binding but no transcriptional activity of the transcription factor *in vitro*. Characterizing the mechanism of cell death after infection with pathological prion protein increased caspase-9 and caspase-3 activity was detected and the lack of NF-κB activity resulted in the inability to activate target genes that usually play an important role in neuroprotection. Additionally, we investigated the role of NF-κB after prion infection of *Nfkb1*^–/–^, *Nfkb2*^–/–^ and *Bcl3*^–/–^ mice and central nervous system-specific p65-deleted mice revealing an accelerated prion disease in NF-κB2- and Bcl-3-deficient mice, which is in line with a reduced neuroprotective activity in prion infection. Based on our findings, we propose a model whereby the alteration of NF-κB activity at the early stages of infection with pathological prion protein leads to neuronal cell death mediated by mitochondrial apoptosis.

## Introduction

Prion diseases represent a group of lethal neurodegenerative disorders, associated with the conversion of the cellular protein PrP^c^ into an abnormally folded isoform, PrP^Sc^. The accumulation of the misfolded protein and the associated extensive neuronal cell loss result in degenerative alterations of the brain as represented by an extensive vacuolation. This is characteristic for transmissible spongiform encephalopathies (TSE) in human and animals. The neuronal cell death in prion disease was described as an apoptotic process (Hetz *et al*., 2003; Cronier *et al*., 2004). However, the signal pathway from the recognition of the pathological agent by the host cell to the nuclear events preceding cell death is not completely understood. The activation of the effector caspase-3 represents a main event in apoptotic processes. This can occur directly upon auto-processing of caspase-8 to its active form after recruitment of death receptors. Another, indirect pathway involves the release of death-promoting factors like cytochrome *c* from the mitochondria. Here, the Bcl-2 family of proteins contains positive (Bax, Bak, Bcl-x_s_, Bad, Bid) and negative regulators (Bcl-2, Bcl-x_L_, Bcl-w) of apoptosis at the mitochondrial level. Both pathways have been described in neurodestructive processes (reviewed in Polster and Fiskum, 2004).

The transcription factor NF-κB plays a central role in the regulation of a large variety of cellular events. Apart from its function as regulator of the expression of inflammatory cytokines, chemokines, immunoreceptors and adhesion molecules, it influences apoptosis in several cell types (reviewed in: Pahl, 1999; Karin and Lin, 2002). Nuclear factor kappa B (NF-κB) represents dimeric transcription factors that belong to the Rel family, which encompass five members: NF-κB1 (p105/p50), NF-κB2 (p100/p52), RelA (p65), RelB and c-Rel. Dimers containing RelA, RelB or c-Rel are transcriptional activators whereas homodimers of p50 and p52, which are devoid of a transcription activation domain, function as repressors. In non-stimulated cells, NF-κB is sequestered in an inactive form in the cytoplasm by the inhibitor of kappaB (IκB). After successive phosphorylation and degradation of IκB, NF-κB translocates to the nucleus and exerts its biological function (reviewed in Bonizzi and Karin, 2004). In the brain NF-κB is an important regulator of biochemical and molecular cascades that can either prevent cell death and promote neuronal plasticity or induce apoptosis. NF-κB is activated by various intercellular signals including cytokines, neurotrophic factors and neurotransmitters. Depending on the cell type the same signal can either induce or prevent apoptosis (Mattson *et al.*, 2000). In addition, alteration of NF-κB activity may represent an important mechanism for inducing or enhancing apoptosis (Barkett *et al*., 1997; Mattson *et al*., 1997). Concerning neuronal cell death, considerable evidence has been presented that NF-κB induces the expression of anti-apoptotic gene products (Mattson *et al*., 2000; Bhakar *et al*., 2002). NF-κB was proposed to protect neurons temporarily from the amyloid β-mediated apoptosis leading to neurodegeneration during Alzheimer's disease (Kaltschmidt *et al*., 1999). Nevertheless, in a mouse model of stroke NF-κB activation enhances neuronal death, where the activation of IKK plays an essential role (Herrmann *et al*., 2005). In prion diseases very little is known on the role of NF-κB concerning neuropathology. An enhanced NF-κB activity has been described in the brain of scrapie-infected mice (Kim *et al*., 1999). Furthermore, the synthetic peptide of the prion protein PrP 106–126 activates NF-κB in microglial cells (Fabrizi *et al*., 2001). In order to elucidate the role of NF-κB in prion infection, we addressed the question whether NF-κB activity is altered after acute infection with the pathological prion protein in a neuroblastoma cell line (Bos2 cells) (Bosque and Prusiner, 2000), and in the mouse model of prion infection. In particular, we were interested in the mechanism of neuronal cell death early after infection with the pathological prion protein. We found an enhanced NF-κB binding activity in infected Bos2 cells, whereas no activation of a NF-κB reporter was observed. Furthermore, we were able to demonstrate an involvement of mitochondria in apoptosis induced early after prion infection. In order to generalize our *in vitro* findings, we also investigated the role of NF-κB *in vivo* after prion infection of *Nfkb1*^–/–^, *Nfkb2*^–/–^ and *Bcl3*^–/–^ mice and a mouse strain, where p65 (RelA) was deleted in the central nervous system (CNS). After intracerebral infection, the *Nfkb2*^–/–^ and *Bcl3*^–/–^ animals appeared to be more sensitive to prion infection and died at an earlier time point compared with wild-type control mice. We hypothesize a loss of protective function of NF-κB in the context of prion disease and propose a possible mechanism where anti-apoptotic genes like *Bclx*_*L*_ are downregulated.

## Results

### NF-κB binding but no transcriptional activity induced by PrP^Sc^

**Fig. 1:**
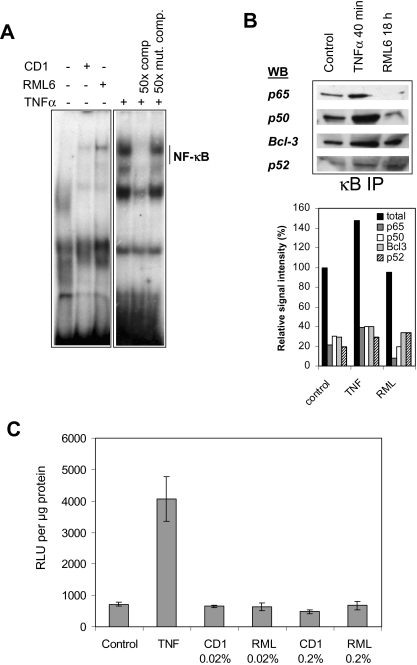
No transcriptional NF-κB activity after PrP^Sc^ infection. A. Electromobility shift assay revealed NF-κB binding activity in PrP^Sc^-infected neuroblastoma cells (Bos2). Nuclear extracts of Bos2 cells infected with RML6, treated with CD1 or stimulated with 10 ng ml^−1^ TNF-α were incubated with ^32^P-labelled double-stranded oligonucleotide specific for NF-κB. The TNF-α sample was incubated with a 50-fold excess of cold NF-κB oligonucleotide as specific competitor or with a mutated oligonucleotide as control. B. κB precipitation experiment and detection using antibodies directed against p65, p50, p52 and Bcl-3. κB binding proteins were precipitated from nuclear extracts using an agarose-conjugated consensus κB oligonucleotide. The precipitates from either untreated controls, TNF-treated or RML6-infected Bos2 cells were divided and analysed in Western blots for the presence of the various NF-κB subunits. The immunoblot was quantified by scanning densitometry. The total signal intensities (p65 + p50, +p52 + Bcl-3) for the treated cells relative to the control cells and the portion of each subunits are given in percentage. C. Luciferase reporter gene assay showed no transcriptional NF-κB activity after PrP^Sc^ infection (0.2% or 0.02%), the value of relative light unit (RLU) per μg protein being equivalent for RML6- and CD1-treated cells. Mean values and SEM of three independent transfections are shown.

The highly PrP^Sc^-susceptible subclone of the N2A neuroblastoma cell line (Bos2) (Bosque and Prusiner, 2000) was used for infection with PrP^Sc^ scrapie strain RML6. Our study was focused on the use of PrP^Sc^ from mouse brain. Therefore, a 0.2% brain homogenate of terminally sick PrP^Sc^-infected CD1 mice were used for infection. As controls, cells were treated with a non-infected brain homogenate (CD1) with equal concentrations. The dilution of brain homogenate used for either *in vitro* or *in vivo* infection was improbably sufficient to induce an unspecific response mediated by factors present in the brain (e.g. cytokines) (Brown *et al*., 2003). After acute PrP^Sc^ inoculation of Bos2 cells infection was controlled by cell blot technique as described in *Experimental procedures* for each experiment (data not shown). To determine DNA binding activity of NF-κB after prion infection, the cell nuclei of PrP^Sc^-infected and control cells were isolated and tested in an electromobility shift assay (EMSA). An increase of NF-κB binding was found as early as 1 day after incubation with RML6 compared with cells treated with non-infected brain homogenate and compared with untreated Bos2 cells (Fig. 1A). Enhanced NF-κB binding was also found after tumour necrosis factor (TNF)-α treatment. This DNA/protein complex disappeared after competition with a 50-fold excess of non-radioactive NF-κB/DNA probe but not with a mutant probe. This control demonstrates the specificity of the signal on the autoradiography. Taken together, these results indicate that RML6 infection induces NF-κB binding activity in Bos2 cells above background levels of normal brain homogenate.

To elucidate the transcriptional activity of NF-κB, luciferase reporter gene assays were performed. Therefore, Bos2 cells were transiently transfected with a κB-responsive reporter construct and 6 h later cells were infected with RML6. After an incubation period of 20 h the cells were lysed and the cell extracts were used for luciferase activity measurement. No activation was found after incubation of Bos2 cells with RML6 compared with CD1-treated or -untreated control cells. However, NF-κB transcriptional activity was observed when Bos2 cells were treated with TNF-α as a positive control (Fig. 1C). These data indicate that the NF-κB binding complex induced by RML6 is not transcriptionally active in Bos2 cells.

To further characterize the nature of the NF-κB dimers presented in the nucleus after acute PrP^Sc^ infection, precipitations of κB binding proteins with an agarose-conjugated consensus κB oligonucleotide were performed and the precipitates were analysed by Western blot (Fig. 1B). While p65 was detected in the nucleus of control and TNF-α-treated cells, almost no signal for p65 was found after RML6 infection. In contrast, p50, Bcl-3 and p52 were precipitated from the nucleus of RML6-infected cells. For further evaluation of these results the blots were quantified by scanning densitometry. While the p65 to p50 ratio reached a value of 0.72 in control cells it decreases to 0.42 after infection. The reduction of the transcriptional active NF-κB subunit p65 supports the formation of inactive homodimers like p50-p50. These results are in line with the lack of transcriptional activity in RML6-treated cells, as p50 and p52 lack a transcription activation domain.

### PrP^Sc^ induces apoptosis via mitochondrial disruption in Bos-2 cells

**Fig. 2:**
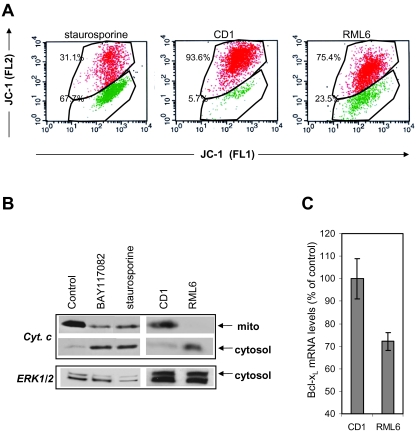
PrP^Sc^-induced mitochondrial disruption leads to apoptosis. A. JC-1 staining of staurosporine, CD1 (control) and RML6 cells. Cells treated with staurosporine (1 μM, 4 h), CD1 or RML6 for 18 h were stained with JC-1 according to the protocol and analysed on a BD FACS Calibur™ as described in *Experimental procedures*. JC-1 fluorescence is seen in both the FL-2 (red) and FL-1 channels (green). JC-1 that fluoresces in the FL-1 channel and lacks fluorescence in the FL2 channel is considered to correspond to mitochondria with a depolarized ΔΨ. For staurosporine-treated cells 67% showed decreased fluorescence in the FL-2, while only a small proportion of the population in CD1 control cells (5.7%) showed depolarization of the ΔΨ. After RML treatment 23.5% of the cells mitochondria were found with a depolarized ΔΨ. B. Western blot analysis of mitochondrial (mito) and cytosol fraction of untreated and either staurosporine-, BAY117082-, CD1- or RML6-stimulated Bos2 cells, using an antibody directed against cytochrome *c*. Equal loading was controlled by detection of ERK1/2 in the cytosol fraction. C. Real time qPCR to detect murine *bcl-xl* from RML6- versus CD1-treated cells. The expression of *bcl-xl* normalized to 18S RNA and the SEM for triplicate experiments are given in percentage of the control (CD1). Statistic analysis using the Mann–Whitney rank sum test showed significant differences between RML6- and CD1-treated cells (*P* = 0.028).

It is well known that PrP^Sc^ induces apoptosis in neurons *in vitro* and *in vivo* (Hetz and Soto, 2003; Cronier *et al*., 2004). Nevertheless, the mechanism of cell death is not completely understood. Mitochondrial dysfunction is a well described event in many cases of apoptosis and has been reported to play a central role in neuropathological processes (Polster and Fiskum, 2004). This pathway is characterized by loss of transmembrane potential (Ψm). Analysis of Ψm using the lipophilic fluorescent dye JC-1 showed an increased number of cells with depolarized Ψm in RML6-treated cells (23.5%) compared with CD1-treated control cells (5.7%), 18 h after RML6 infection (Fig. 2A).

The release of cytochrome *c* from the mitochondrial intermembrane space into the cytosol commonly follows membrane depolarization. In order to determine the localization of cytochrome *c*, mitochondrial and cytosolic fractions of cells 18 h after incubation with RML6 or normal brain homogenates were prepared. Staurosporine-treated cells were used as positive control and the NF-κB inhibitor BAY117082 was tested for its ability to induce cytochrome *c* release. Using Western blot analysis cytosolic cytochrome *c* was detected in staurosporine-, BAY117082- and RML6- but not CD1-treated cells (Fig. 2B). Consequently, our data show that infection of Bos2 cells with RML6 leads to an early induction of a decrease in the mitochondrial membrane potential, followed by cytochrome *c* release into the cytosol.

The mitochondrial permeabilization and the release of death-promoting factors into the cytosol depend on the regulation by proteins of the Bcl-2 family (Kroemer, 1997). To further investigate the apoptotic pathway a cDNA microarray analysis was performed. Bos2 cells were inoculated with RML6 and RNA was extracted at 8, 18 and 30 h after treatment. CD1-treated Bos2 cells were used as controls. To assure biological relevance three independent experiments were performed, where the RNA from cells was extracted. The PIQOR™ Cell Death Microarray (Memorec) was used composed of genes encoding key proteins in apoptosis, stress-like caspases, TNF-receptor family members, Bcl-2 family members and heat shock proteins. Various genes showed different expression patterns at 8, 18 and 30 h after treatment and most of them were significantly regulated only at 18 h. At this time point 21 transcripts were differentially regulated. Their level of regulation in RML6-treated cells compared with control cells is summarized in Table 1. Interestingly among the 13 downregulated genes, five (*Tnfrsf11a/Rank*, *CcnD1*, *Myc*, *Junb* and *Bclx*_*L*_) are known to be NF-κB targets (Pahl, 1999). As Bcl-x_L_ is involved in the regulation of mitochondrial apoptosis, the differential expression of Bcl-x_L_ was further confirmed using real time quantitative PCR (qPCR). Here, a 28% downregulation of Bcl-x_L_-specific mRNA in RML6-infected cells compared with control cells was observed (Fig. 2C). This finding that Bcl-x_L_-specific mRNA is downregulated in Bos2 cells after acute PrP^Sc^ infection perfectly correlates with the mitochondrial dysfunction and the cytochrome *c* release.

**Table 1:** Differential gene expression in Bos2 + RML6 versus Bos2 + CD1 18 h p.i.

Name	Gene ID	SWISSPROT	Function	Fold/deviation
Upregulated				
Lyar	22757	Q08288	Regulation of cell growth	1.82/11%
Chmp5	17765	Q9D7S9	Protein transport	1.82/11%
Fas	78	Q9DCQ1	Receptor for TNFSF6/FASLG	1.82/8%
MKi67	1222	Q61769	Antigen	2.06/18%
Hsp90aa1	1719	P07901	Heat shock protein	1.74/3%
Hsp105	3581	Q61699	Heat shock protein	1.79/13%
Trp53bp2	8225	Q8K2L5	Regulation of cell growth and apoptosis	1.74/13%
IFi203	18178	O353668	Interferon (IFN)-α-inducible protein	1.82/–%
Downregulated				
Cdkn1B	90	P46414	Cell cycle regulation	0.53/13%
CcnD1^a^	123	P25322	Cell cycle regulation	0.50/7%
Ngfr	235	Q9Z0W1	Regulation of cell survival as well as cell death of neural cells	0.46/3%
Tnfrsf11a^a^	244	Q8VCT7	Receptor for TNFSF11/RANKL	0.35/18%
Bcl2l13	269	P59017	Apoptose regulator	0.52/13%
Junb^a^	3865	Q8C2G9	Transcription factor	0.56/13%
Tyk2	4106	Q8VE41	Signal transduction of type I and type II IFN	0.58/0%
Myc^a^	4192	P01108	Regulation of gene transcription	0.58/6%
Tcfe3	4260	Q8K420	Transcription factor	0.46/6%
Enc1	8555	O35709	Actin binding protein	0.26/5%
Bcl-X^a^	64	Q64373	Apoptosis inhibitor	0.58/13%
Tollip	18989	Q9QZ06	IL-1 and Toll-like receptors signalling	0.53/9%
Ticam1	21115	Q8JZV0	Toll-like receptors signalling	0.56/8%

a Indicates NF-κB target genes.

Taken together, our data show that RML6 induces mitochondrial permeabilization and a decrease in mitochondrial membrane potential in Bos2 cells. Moreover, RML6 leads to downregulation of individual NF-κB target genes, among them the anti-apoptotic regulator Bcl-x_L_.

### PrP^Sc^ activates caspase-9 and caspase-3 in Bos-2 cells

**Fig. 3:**
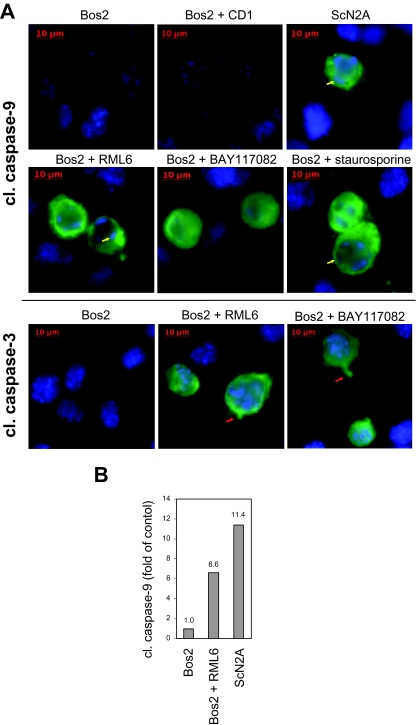
PrP^Sc^ induced the activation of caspase-9 and caspase-3. A. Detection of active caspase-9 and caspase-3 in untreated Bos2 and ScN2a cells and in Bos2 cells treated with CD1, RML6, BAY117082 or staurosporine for 20 h. The active caspase staining is shown in green and the nuclear DAPI staining is shown in blue. Cells presenting chromatin condensation are indicated with yellow arrows and blebs with red arrows. B. Quantitative analysis of active caspase-9 in Bos2, RML6-infected Bos2 and ScN2A cells. Cleaved caspase-9-positive cells were given in percentage of control (untreated Bos2 cells). The cells from one of three representative experiments per 50 mm^2^ were counted.

Release of cytochrome *c* leads to specific activation of caspase-9. Therefore, we investigated the expression of this caspase in Bos2 cells. No caspase-9 activation was found in untreated Bos2 cells or in CD1-treated cells, whereas caspase-9 activation was found in persistently infected N2A cells (ScN2A) (Fig. 3A). Positive cells for active caspase-9 were also found after RML6 infection of Bos2 cells and after treatment of these cells with either BAY117082 or staurosporine. Moreover, these caspase-9-positive cells showed chromatin condensation (yellow arrows), which is a characteristic of cells undergoing apoptosis. To further support these findings a quantitative analysis of the fluorescence staining was performed as described in the figure legend. A 6.6-fold increase of active caspase-9-positive cells was found after RML6 infection of Bos2 cells and a 11.4-fold increase in persistently prion-infected N2A cells compared with Bos2 cells (Fig. 3B). In contrast to N2A/Bos2 cells, GT1 cells were not suitable for these experiments because of a high caspase-9 activity in untreated cells (Fig. S1).

To further investigate the apoptotic events after acute infection of Bos2 cells with RML6, fluorescence staining was also performed for the detection of activated caspase-3. While no caspase-3 activity was found in uninfected Bos2 cells, activated protein was detectable in RML6-infected Bos2 cells and in Bos2 cells treated with the NF-κB inhibitor BAY117082 as a positive control. The caspase-3-positive cells presented membrane blebbing (red arrows), a hallmark of apoptosis (Glasgow and Perez-Polo, 2000).

### PrP^Sc^ infection of mice lacking NF-κB1, NF-κB2 and Bcl-3

**Fig. 5:**
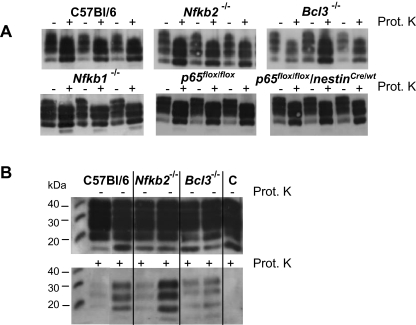
Western blot analysis revealed equal amounts of PrP. A. Western blot analysis of brains homogenates subjected to electrophoresis in native form (–) or after digestion with proteinase K (+) showed nearly similar amounts of PK-resistant PrP^Sc^ in knockout and wild-type mice that had developed terminal disease. B. Analysis of brains homogenates at 112 days p.i. revealed comparable amounts of prion protein. Variation between individual animals for PK-resistant PrP^Sc^ was observed in wild-type and NF-κB-deficient mice. C indicated a control performed with non-infected brain homogenate of C57Bl/6 mice, where no prion protein can be detected after PK treatment.

To investigate whether NF-κB is involved in prion pathogenesis *in vivo*, we used mice lacking either p65 or p50, compounds of the classical NF-κB pathway, or mice lacking p52 or Bcl-3 for infection with PrP^Sc^. In order to use *p65*^flox/flox^/*nestin*^Cre/wt^, *Nfkb1*^–/–^, *Nfkb2*^–/–^ and *Bcl3*^–/^ mice for our study, we first investigated if all strains showed comparable expression of cellular PrP in the brain. Western blot analysis indeed revealed comparable levels of PrP^c^ in all mouse strains tested (Fig. 5, data not shown).

**Fig. 4:**
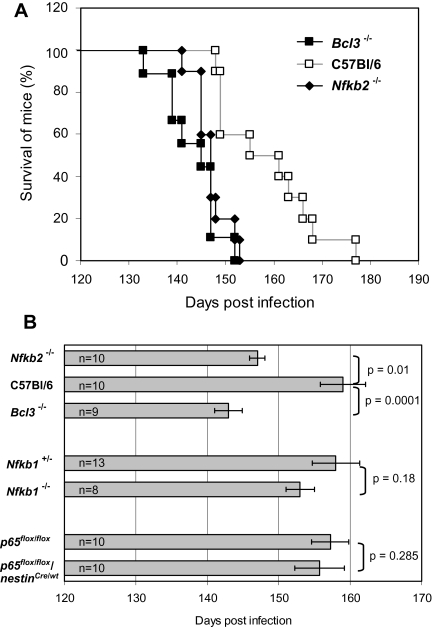
Terminal disease in NF-κB-deficient mice after PrP^Sc^ infection. A. Kaplan Meier survival curves of wild-type, NF-κB2- and Bcl-3-deficient mice. *Bcl3*^–/–^, *Nfkb2*^–/–^ and C57Bl/6 mice as control were inoculated intracerebrally with 30 μl of 0.2% (w/v) RML6 brain homogenate, monitored daily for clinical symptoms and euthanized at the end-point of neurological disease. B. Mean of survival and SEM for all mice lines analysed are represented. Terminal disease stage is significantly reduced in NF-κB2-deficient (*P* = 0.01) and Bcl-3-deficient (*P* = 0.0001) mice compared with C57Bl/6 mice. NF-κB1-deficient mice showed no difference in the time-course of the disease compared with relevant heterozygous controls (*P* = 0.18). See *Experimental procedures* for detailed description of mice stains. Mice with p65 deletion in nestin expressing cells (*p65*^*flox/flox*^*/Nestin*^*Cre/wt*^) showed no difference in disease development compared with mice with intact p65 expression (*p65*^*flox/flox*^) (*P* = 0.285). Number of mice (n) and *P*-value are given.

Wild-type C57Bl/6 mice infected with RML6 developed disease around day 136 post infection (p.i.) and became terminal sick by day 158 ± 10.1. Mice lacking NF-κB2 already developed disease by day 120 and were terminally sick by day 147 ± 3.5. Bcl-3-deficient mice showed first disease symptoms around day 128 p.i. and terminal disease by day 143 ± 5.8 after infection. This demonstrated a significant increase in onset of terminal disease in Bcl-3-deficient (*P* = 0.0001) and NF-κB2-deficient (*P* = 0.01) mice. As the *Nfkb1*^–/–^ mice showed an unspecific background, *Nfkb1* heterozygous littermates were used as controls. These animals developed disease after RML6 infection at time points comparable to C57Bl/6 mice (onset 143, terminally sick 158 ± 11.9). When *Nfkb1*^–/–^ mice were inoculated with RML6 they showed first signs of disease by day 143 p.i. and the end stage of disease at 153 ± 5.6 days after infection. Thus, there is no significant difference (*P* = 0.18) of incubation time in mice lacking NF-κB1 compared with controls (Fig. 4 and Fig. S2). To further elucidate the role of the classical NF-κB pathway we infected p65-deficient mice. Tissue-specific p65-deficient mice were generated by crossing *p65*^*flox/flox*^ mice with CNS-specific *nestin-Cre* mice. *p65*^*flox/flox*^*/Cre*-negative animals have a normal p65 expression in the brain and developed disease after RML6 infection at time points comparable to C57Bl/6 mice (onset 141, terminal stage of disease 157 ± 8.3, *P* = 0.62). The *Cre*-positive mice (*p65*^*flox/flox*^*/nestin*^*Cre/wt*^) with p65 deletion in the CNS showed no difference to their control littermates (onset 141, terminal stage of disease 156 ± 11.0, *P* = 0.28).

### Spongiform encephalopathy and expression of Bcl-x_L_, active caspase-9 and active caspase-3 in NF-κB-deficient mice after PrP^Sc^ infection

**Fig. 6:**
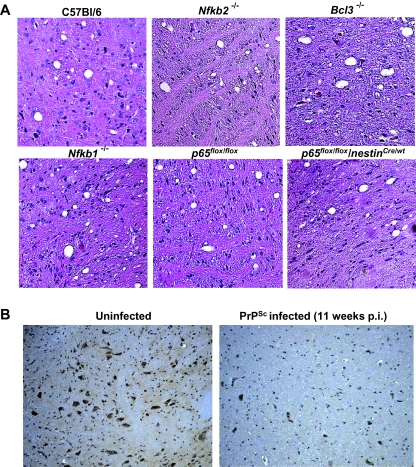
Lesions in the brain and p65 detection of PrP^Sc^-infected mice. A. Terminally sick mice showed comparable vacuolation (haematoxylin/eosin staining, H&E) after i.c. inoculation of PrP^Sc^. The pons areas of midsagittal sections are shown. Magnification ×20. Three mice of each strain were analysed. B. Strong reduction of activated NF-κB (phospho-p65) in PrP^Sc^-infected C57Bl/6 mice. Uninfected and infected animals at 11 weeks p.i. were sacrificed and the brain of three animals per group were analysed by immunohistological staining using a phospho-p65-specific antibody. Representative pons areas of midsagittal sections are shown. Magnification ×10.

In experimental prion disease of mice, the quantity of PrP^Sc^ in the brain and the neuropathological changes after infection characterized by spongiform encephalopathy can not always be correlated with clinical symptoms or onset of disease (Hill *et al*., 2000). In C57Bl/6 mice PrP^Sc^ deposition and spongiosis was predominantly found in the cerebellum, pons, entorhinal cortex, medulla and to lower extends in the thalamus. In Bcl-3- and NF-κB2-deficient mice the amount and the distribution of PrP^Sc^ was comparable to the C57Bl/6 wild-type control mice as demonstrated by immunohistochemistry (data not shown) and Western blot analysis (Fig. 5A). Analysis of control and NF-κB-deficient animals at the exact same day post infection (112 p.i.) revealed comparable amount of PrP^Sc^ too, albeit variation of the signal after protease K digestion between the individual animals was observed in all panels (Fig. 5B). More interestingly, the level of vacuolation in the terminal stage of disease was also comparable to the wild-type animals, even though the onset of disease in NF-κB2- and Bcl-3-deficient mice was earlier (Fig. 6A).Active p65 is present in the CNS (Kaltschmidt *et al*., 1995). According to our *in vitro* data (Fig. 1), we now raised the question, whether the level of active p65 is altered in the CNS of mice after PrP^Sc^ infection. While in uninfected C57Bl/6 mice the presence of active p65 is clearly demonstrated, a reduction of active p65 was found in mice 11 weeks after PrP^Sc^ infection (Fig. 6B).

**Fig. 7:**
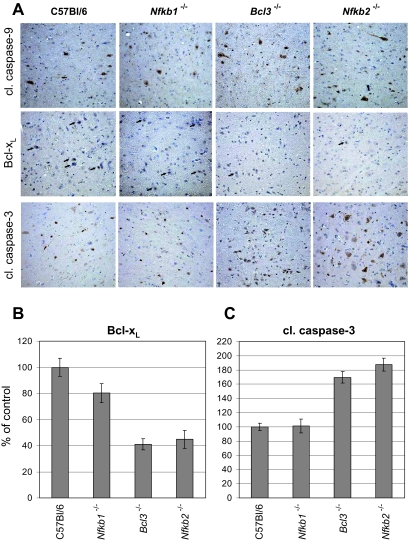
Reduced Bcl-x_L_ expression correlates with enhanced caspase-3 activation in NF-κB2- and Bcl-3-deficient mice after 11 weeks of PrP^Sc^ infection. A. Bcl-x_L_ expression (black arrows), active cleaved caspase-9 and caspase-3 were determined by immunohistological staining. *Bcl3*^–/–^, *Nfkb2*^–/–^, *Nfkb1*^–/–^ and C57Bl/6 mice were inoculated with RML6 and three animals per groups were sacrificed at 11 weeks p.i. Brain sections of three animals were analysed for each mouse line. Representative areas of the entorhinal cortex are shown. Magnification ×20. B and C. For quantification of positive cells, three sections of three different mouse brains for each strain were chosen. The graphic represents the amount of positive cells in the brain of the deficient mice compared with the C57Bl/6 mice in percentage of control. SEM was shown as bars. For detailed information on quantification see *Experimental procedures*.

Our *in vitro* experiments demonstrated that prion infection resulted in mitochondria-mediated apoptosis associated with reduction of Bcl-x_L_ specific mRNA expression, cytochrome *c* release and activation of caspase-9 and caspase-3. We now questioned if this mechanism of cell death might be also functional in the *in vivo* situation. Mice were infected intra-cerebral (i.c.) with RML6 and the brains of these animals were investigated by immunohistochemistry at various time points after infection until the terminal disease stage. At late stages of the disease, when neurodegeneration becomes obvious, no differences in the expression of Bcl-x_L_ in C57Bl/6 mice compared with *Nfkb1*^–/–^, *Nfkb2*^–/–^ and *Bcl3*^–/–^ mice were found (data not shown). An increase of Bcl-x_L_-positive cells in RML6-infected C57Bl/6 mice compared with uninfected animals was found at 11 weeks after infection, while a reduced number of Bcl-x_L_-expressing cells was found in the brain of NF-κB2- and Bcl-3-deficient mice compared with C57Bl/6 mice at the same time point (Fig. 7A). Interestingly, in NF-κB1-deficient mice Bcl-x_L_ staining was comparable to C57Bl/6 mice. These observations were supported by quantitative analysis of the brain section (Fig. 7B). Roughly, a 60% reduction of Bcl-x_L_-positive cells was found in the brain of NF-κB2- and Bcl-3-deficient mice compared with C57Bl/6 mice.

Activation of caspase-9 is involved in the major mitochondrial apoptotic pathway. By immunohistochemistry activation of caspase-9 was found in the brain of prion-infected mice but only slight differences between the various mouse strains concerning the number of positive cells were present (Fig. 7A). This was most probably due to high caspase-9 expression in uninfected mouse brains. Therefore, in addition, immunohistological analysis of activated caspase-3 was performed. Activation of caspase-3 by cleavage of the pro-caspase-3 is a central step to induce apoptosis. Detection of active caspase-3 is a solid and useful marker to investigate apoptosis. By immunohistochemistry, even in a normal adult mouse brain active caspase-3-positive cells can be found in particular in the olfactory bulb and single positive cells can be detected throughout the brain. The picture of active caspase-3-positive cells totally changes in PrP^Sc^-infected mice at terminal disease stage. Here, intensive active caspase-3 staining can be found basically in all areas where spongiform vacuolation is found, and to lower extends also in areas of the brain without obvious neuropathological changes. When brains of either terminally diseased wild-type or NF-κB-deficient mice were analysed in immunohistochemistry with an antibody directed against active caspase-3, no differences in the amount and the distribution of active caspase-3 was found (data not shown). Therefore, we investigated brain sections at earlier time points after PrP^Sc^ infection. According to the results of Bcl-x_L_ expression, brain sections were tested for caspase-3 activity at 11 weeks p.i. Here, we found increased caspase-3 activity in NF-κB2- and Bcl-3-deficient mice compared with C57Bl/6 and NF-κB1-deficient mice (Fig. 7A). Quantitative analysis of caspase-3-positive cells revealed a 65% increase in Bcl-3-deficient mice and an 82% increase in NF-κB2-deficient mice (Fig. 7C). The reduction of the anti-apoptotic protein Bcl-x_L_ correlates with the increased activation of caspase-9 and more obvious to an increased activation of caspase-3 that consequently leads to apoptosis.

## Discussion

A notable feature in prion disease is a progressive loss of neurons, gliosis and spongiform changes. The cellular mechanisms of nerve cell death is still poorly understood, but it has been shown that apoptosis plays a major role (Giese *et al*., 1995; Lucassen *et al*., 1995; Hetz *et al*., 2003). Our data suggest that NF-κB is involved in early events leading to apoptosis and show that apoptosis might not be the only factor involved in the pathology of the disease.

How can NF-κB's mode of action be envisioned? Activation of the transcription factor κB in the central nervous system prevents neuronal apoptosis in cell culture and in animal models (Barger *et al*., 1995; Kaltschmidt *et al*., 1995; Mattson *et al*., 1997). Several studies have shown that neuronal dysfunction after prion infection occurs through a process of programmed cell death. This is true in the murine experimental models for scrapie but also for Creutzfeldt-Jakob Disease (CJD) in humans (reviewed in Hetz and Soto, 2003). Apoptosis was also found in primary cultured neurons, in a hypothalamic neuronal cell line and in neuroblastoma cells (Barger *et al*., 1995; Hetz *et al*., 2003; Cronier *et al*., 2004; Kristiansen *et al*., 2005). Our experiments demonstrated that after acute prion infection of Bos2 neuroblastoma cells with PrP^Sc^ (RML6), NF-κB binding activity was detectable but no transcriptional activity. This obvious discrepancy could be solved when the subunits of the NF-κB complex were identified to demonstrate that p65 did not translocate to the nucleus of RML6-infected Bos2 cells. Furthermore, *in vivo* a reduced expression of phospho-p65 was found in PrP^Sc^-infected C57Bl/6 mice at early stages of the disease (11 weeks) compared with uninfected mice. In contrast to p65, the NF-κB proteins p50 and p52 lack a transactivation domain and cannot achieve for the activation of transcription. Homodimeric complexes of p52-p52 or p50-p50 are associated with repression of transcription (Bours *et al*., 1993; Kastenbauer and Ziegler-Heitbrock, 1999). In fact, we observed a downregulation of NF-κB target genes like RANK, cyclinD1 and Bcl-x_L_ in acute PrP^Sc^-infected cells (Pahl, 1999; Shishodia *et al*., 2005). This is in agreement with results obtained from persistently infected ScN2A cells, where various genes involved in translation or protein synthesis were downregulated (Greenwood *et al*., 2005). Our data on NF-κB DNA binding activity after PrP^Sc^ infection are supported by the work of Fabrizi *et al*. (2001) which described NF-κB activity in human microglial cells after stimulation with the prion protein fragment 106–126. Furthermore, NF-κB activity has been found in brains of scrapie-infected mice at the terminal state of the disease (Kim *et al*., 1999).

One might now raise the question whether the repression of NF-κB activity participates to neuronal cell death after PrP^Sc^ infection. Our data support the view that PrP^Sc^ leads to mitochondrial dysfunction, cytochrome *c* release, activation of caspase-9 and caspase-3 and subsequently to apoptosis. In neuropathological processes mitochondrial dysfunction is often present, and it is well known that mitochondria are involved in neuronal cell apoptosis (Polster and Fiskum, 2004). Cytochrome *c* released from mitochondria together with other proteins leads to the production of active caspase-9 that again induces caspase-3 activation and results in apoptosis (Shi, 2002). Furthermore, our data support earlier findings where the synthetic peptide PrP (106–126) of prion protein was able to induce cell death by mitochondrial disruption in neuronal cells (O'Donovan *et al*., 2001).

In the last decade, members of the Bcl-2 protein family have emerged as important regulators of the mitochondrial membrane potential (Ψm). Their exact mechanism is yet not completely understood but it is a general hypothesis that anti-apoptotic Bcl-2 inhibits the opening of pores at the mitochondrial outer membrane and thereby prevents the loss of Ψm, which consequently prevents the release of cytochrome *c* and apoptosis (Kroemer, 1997). In our microarray analysis we found downregulation of Bcl-x_L_ after acute infection of Bos2 cells. The death antagonist Bcl-x_L_ is a member of the Bcl-2 protein family. Interestingly, *Bclx*_*L*_ was reported to be an NF-κB target gene (Pahl, 1999). Other inhibitors of apoptosis proteins (IAP, XIAP, NAIP) or FLIP block the activation of caspases, particularly those involved in engagement of the receptor-related, extrinsic apoptotic pathway (Karin and Lin, 2002). In our microarray analysis we found no differences in the regulation of IAPs. One therefore might argue that apoptosis after acute PrP^Sc^ infection is predominately regulated by the intrinsic, mitochondrial apoptotic pathway. Using N2A cells stably expressing mutant form of Bcl-2, it has been described that Bcl-2 regulates the induction of apoptosis mediated by endoplastic reticulum (ER) stress rather than by mitochondrial dysfunction after PrP^Sc^ infection (Hetz *et al*., 2003). We cannot rule out that Bcl-x_L_ exerts another function than Bcl-2 and it could be possible that in addition to the mitochondial dysfunction, PrP^Sc^ induced cell death over ER stress and caspase-12 activation. Nevertheless, in permanently PrP^Sc^-infected hypothalamic neurons (Sc-GT1 cells) an apoptotic pathway involving formation of aggresomes together with the activation of caspase-8 and caspase-3 was described (Kristiansen *et al*., 2005). While these cells contain high levels of prion protein, this cell culture model may rather reflect the situation at the late stage of the disease, whereas the NF-κB repression and the involvement of mitochondria leading to neuronal apoptosis within 24 h in acute infected Bos2 (N2A) cells may represent an early event after PrP^Sc^ infection.

Consequently the question now arises, if our findings based on cell culture experiments also reflect the situation *in vivo*. To analyse various NF-κB signalling pathways we used NF-κB1-, NF-κB2- and Bcl-3-deficient mice and animals with p65 depletion in the CNS for infection with PrP^Sc^. Except the p65^–^/^–^ line, these deficient mice have severe defects in their lymphoid organs (reviewed in Bonizzi and Karin, 2004), which are important sites of prion replication upon intra-peretonial (i.p.) prion inoculation. As we exclusively performed i.c. inoculation these mouse strains were adequate for our studies. Interestingly, the enhanced susceptibility of NF-κB2- and Bcl-3-deficient animals compared with NF-κB1-deficient mice and wild-type controls correlates with a reduced expression of the anti-apoptotic protein Bcl-x_L_ 11 weeks after prion infection. This is in line with our *in vitro* results, where Bcl-x_L_ mRNA was downregulated in acute PrP^Sc^-infected cells. Furthermore, enhanced cleaved caspase-3 activity was found in NF-κB2- and Bcl-3-deficient mice at the same time point. The different patterns of caspase-3 expression and the reduced level of Bcl-x_L_ in the NF-κB2- and Bcl-3-deficient animals argue for a Bcl-x_L_-dependent protection in the wild-type mice at early stages of the disease. This is in line with the previously reported neuroprotective role of NF-κB (Glasgow and Perez-Polo, 2000; Dhantapani *et al*., 2005). The involvement of the mitochondrial apoptotic pathway in mice suffering from prion disease is under debate. Bax-deficient mice inoculated with a mouse-adapted BSE strain, presented the same pathology compared with control animals (Coulpier *et al*., 2006). Recently, both Bax-dependent and Bax-independent neurotoxic pathways were found in mice with N-terminally deleted forms of the prion protein (Li *et al*., 2007).

Taken together, our data provide new insides into the mechanism of neuronal cell death early after prion infection. By a so far unknown reason the NF-κB subunit p65 is reduced in early stages after PrP^Sc^ infection. The NF-κB p50 homodimer that lacks transcriptional activity may bind to the promoter region, leading to downregulation of genes coding for anti-apoptotic proteins like Bcl-x_L_. From previous studies it is known that Bcl-3 can function as coactivator for p50 and p52 homodimers (Bours *et al*., 1993; Bonizzi and Karin, 2004). Nevertheless, the role of Bcl-3 in the CNS is almost completely unidentified. A strong signal for Bcl-3 was found in the nucleus of PrP^Sc^-infected Bos2 cells. Therefore, one might speculate that Bcl-3 reverses the repression and induces gene expression. This hypothesis is supported by the earlier onset and terminal stages of disease in mice with either disrupted *Bcl-3* or *Nfkb2* genes. As prion infection of wild-type mice and NF-κB-deficient mice equally resulted in death, one is tempted to argue about the contribution of NF-κB and apoptosis in prion disease. From our and from other studies it is clear that NF-κB activity is not the only factor leading to disease. At a late time point of the disease microglia and astrocytes are activated and produce chemokines and cytokines, which have been associated with the death of the surrounding neurons (Mallucci *et al*., 2003; Bate *et al*., 2004). These chemokines and cytokines probably induce apoptosis via a direct pathway independent of mitochondria and Bcl-x_L_ expression. This is in line with our finding of strong caspase-3 activation in all animals in the terminal stage of the disease. In addition, a neuroprotective mechanism against prion neurotoxicity was described, involving the early expression of the ER chaperones Grp58, which counterparted cell death after stress (Hetz *et al*., 2005). Nevertheless, the findings that in early stages of the developing disease mitochondrial defence mechanisms are involved in neuroprotective processes brings important aspects to the understanding of the development of prion disease. Interestingly, Bcl-x_L_ and other anti-apoptotic proteins were induced by estrogen, a hormone that can protect neurons against neurodegenerative processes and which therapeutic application is considered for prevention or treatment of neurodegenerative disorders like Alzheimer's disease (reviewed in Nilsen and Brinton, 2004). Targeting mitochondrial function would be an approach to achieve neuron viability and reflects new defence strategies against neurodegenerative diseases.

## Experimental procedures

### Mice

NF-κB1-deficient mice with an unspecified background (129-derived E14 ES cell line was used) were purchased from the Jackson Laboratory. All mice were bred and housed in a conventional animal facility at the Friedrich-Loeffler-Institut. Because of the unspecified background *Nfkb1*^–/–^ mice were crossed with C57Bl/6 mice. The heterozygous offsprings were crossed with *Nfkb1*^–/–^ mice resulting in 50% *Nfkb1*^–/–^ mice and 50% *Nfkb1*^+/–^ mice. The *Nfkb1*^+/–^ littermates functioned as controls. Generation of *Bcl3*^–/–^ and *Nfkb2*^–/–^ mice was reported elsewhere. In brief, exons 3–5 and a part of exon 6 from the *Bcl3* gene were replaced by a neomycin cassette (Riemann *et al*., 2005). In NF-κB2-deficient mice exons 1b−9 were replaced (Paxian *et al*., 2002). *Bcl3*^–/–^ and *Nfkb2*^–/–^ mice were backcrossed into C57Bl/6 strain at least 10 times. As the p65 knockout is lethal, tissue-specific animals were generated. The Cre–loxP recombination system was used to excise exons 7–10, coding for a part of the Rel homology domain and the nuclear localization sequence (NLS) of the *p65* gene. The *nestin-cre* mice are published (Betz *et al*., 1996) and *p65*^*flox/flox*^ mice will be described elsewhere (R.M. Schmid, manuscript in preparation). Intercrossing of the *p65*^*flox/flox*^ mouse line with mice carrying *nestin*^*Cre/wt*^ leads to a *p65*^*flox/flox*^*/nestin*^*Cre/wt*^ mouse line with a CNS-specific deletion of p65 and *p65*^*flox/flox*^ control littermates. All mice were healthy, fertile and born at the expected mendelian ration. Conditional deletion of *p65* in neuronal cells was confirmed by Southern blot analysis and is shown elsewhere (Inta *et al*., 2006).

### Cell lines

The mice cell line N2A was subcloned and cells highly susceptible for PrP^Sc^ were described as Bos2 cells and used as a standard laboratory cell line for infection (Bosque and Prusiner, 2000). Permanently PrP^Sc^-infected N2A cells (ScN2A) were kindly provided by Dr Aguzzi (Zürich) and Dr Korth (Düsseldorf). Presence of PrP^Sc^ was controlled on a regular basis using cell blot technique. Cells were cultured in Iscove's modified Eagle's medium supplemented with 10% fetal calf serum, 2 mM l-glutamine and 100 U ml^−1^ gentamicin. Cells were maintained at 37°C in 5% CO_2_.

### Prion inoculations

RML6 scrapie strain was used throughout these experiments and was produced in the Friedrich-Loeffler-Institut in collaboration with Dr Aguzzi, Zürich. The inocula were prepared from brain tissue homogenized in steril 0.32 M sucrose at 20% (w/v). The mice were inoculated intracerebrally with 30 μl of 0.2% (w/v) RML6 brain homogenate or normal brain homogenate from CD1 control mice. Inoculated animals were monitored daily for clinical symptoms and euthanized at the end-point of neurological disease and dysfunction if not otherwise mentioned. For infection of Bos2 cells 20% RML6 or CD1 brain homogenate was added to the culture media at a 1/100 (0.2%) or a 1/1000 (0.02%) ratio (v/v). Cells were tested for the presence of PrP^Sc^ using the cell blot technique according to Bosque and Prusiner. In brief 1.5 × 10^5^ Bos2 cells were seeded in two wells of a 6-well culture plate and infected at the next day. After 24 h in the presence of the inoculum the cells were splitted at a 1:5 ratio in normal medium. Cell blotting was performed at 7 days post inoculation after three times splitting in new plates to remove the initial brain inoculum. By the last passage 1 × 10^4^ Bos2 cells were seeded on glass coverslips and were transferred to the nitrocellulose membrane. To detect PrP^Sc^ cell blot membrane was treated with 5 μg ml^−1^ proteinase K prior to prion detection.

### EMSA

For preparation of nuclear extracts, 2 × 10^5^ Bos2 cells were seeded in 60 mm cell culture dishes and were infected at the next day with RML6 or CD1 (0.02%) or treated with 10 ng ml^−1^ recombinant human TNF-α for 40 min (ImmunoTools). Cells were washed twice with cold PBS and harvested in 400 μl of buffer A (10 mM KCl; 10 mM HEPES, pH 7.9; 0.1 mM EDTA; 0.1 mM EGTA; 1 mM DTT and 1 mM PMSF). After 5 min at 4°C, the cells were passed 10 times through syringe needles (26 gauge). The nuclei were pellet and resuspended in 50 μl of buffer B (20 mM HEPES, pH 7.9; 0.4 M NaCl; 1 mM EDTA; 1 mM EGTA and 1 mM DTT). After 20 min of shaking and subsequent centrifugation, the protein concentration of nuclear extracts was determined (Bio-Rad). Eight micrograms of nuclear extract were incubated with 4 μg of poly(dI-dC)^2^ in binding buffer (0.1 M KCl; 10 mM Tris/HCL, pH 7.5; 5 mM MgCl_2_; 1 mM DTT and 10% glycerol). After 20 min, 2–5 × 10^4^ c.p.m. of ^32^P-labelled oligonucleotides (5′-GATCCAGAGGGGACTTTCCGAGTAC-3′) were added to the mixture and further incubated for 10 min at room temperature. Alternatively, the NF-κB consensus oligonucleotide (5′-AGTTGAGGGGACTTTCCCAGGC-3′) and the mutant oligonucleotide (5′-AGTTGAGGCGACTTTCCCAGGC-3′) as controls for specific competition with unlabelled DNA probes were used. The samples were separated on a no denaturing 5% polyacrylamide gel. After drying the gel was subjected to autoradiography.

### Precipitation of NF-κB binding proteins

For precipitation of NF-κB binding proteins in nuclear extracts, an agarose-conjugated consensus κB oligonucleotide (GGGGACTTTCCC) was used (Santa Cruz Biotechnology). Bos2 cells (1 × 10^6^) were infected with RML6 (0.2%) or treated with 10 ng ml^−1^ TNF-α for 40 min. After 18 h nuclear proteins from control and stimulated Bos2 cells were isolated as described for EMSA. Nuclear extracts from 1 × 10^6^ Bos2 cells were incubated for 2 h at 4°C with 25 μl of beads in 400 μl of κB buffer (20 mM HEPES, pH 7.9; 50 mM NaCl; 0.5 mM EDTA and 1 mM DTT). The precipitated proteins were washed three times and subjected to analysis by SDS-PAGE and Western blot. The membranes were blocked with 1% BSA and 1% non-fat milk in TBST (10 mM Tris/HCl, pH 8.0; 150 mM NaCl and 0.05% Tween 20) for 1 h. For detection antibody against p52 (sc-7386x), p50 (sc-114x) (Santa Cruz Biotechnology), p65 (14-6731, ebioscience) were diluted 1/1000 and the anti-Bcl-3 antibody (sc-185) was diluted 1/200 in blocking buffer. After incubation overnight at 4°C, the membranes were washed three times with TBST and incubated with HRP-conjugated goat anti-rabbit IgG (7074, Cell Signaling) or anti-mouse IgG (sc-2005) for 1 h. Immune-reactive proteins were visualized by enhanced chemoluminescence (ECL) detection system (Santa Cruz Biotechnology).

### Luciferase reportergene assay

Bos2 cells (5 × 10^4^) were plated in 24-well plates and were transfected with 0.05 μg of 3×κB reporter plasmid and 0.2 μl of Lipofectamin^TM^ 2000 (Invitrogen). After 6 h cells were either untreated (control) or treated with TNF-α 10 ng ml^−1^, RML6 or CD1 (0.2% or 0.02%). Twenty-four hours after transfection, the luciferase activity of 20 μl of cleared lysate was quantified in a luminometer (Lumat LB 9501) as described previously (Bourteele *et al*., 2005). For normalization protein concentrations were determined by the Bradford method (Bio-Rad).

### Mitochondrial membrane potential

Bos2 cells in a concentration of 5 × 10^5^ in 5 ml of culture medium were seeded in 60-mm cell culture dishes and inoculated at the next day with RML6 or CD1 (0.2%) or treated with 1 μM staurosporine (Sigma) for 18 h. For determination of mitochondrial potential the cells were stained with JC-1 for 15 min at 37°C according to the instruction of the manufacturer and analysed by flow cytometry (MitoScreen Kit, BD bioscience). When Bos2 cells were incubated with JC-1, JC-1 penetrates the plasma membrane of cells as monomers. Uptake of JC-1 into mitochondria is driven by the ΔΨ. The ΔΨ of normal, healthy mitochondria is polarized and JC-1 is rapidly taken up by such mitochondria. This uptake increases the concentration gradient of JC-1 leading to the formation of JC-1 aggregates within the mitochondria. JC-1 aggregates show a red spectral shift resulting in higher levels of red fluorescence emission which is measured in the Red (FL-2) channel. JC-1 does not accumulate in mitochondria with depolarized ΔΨ and remains in the cytoplasm as monomers. These monomers do not have the red spectral shift, and therefore have lowered fluorescence in the FL-2 channel.

### Extraction of mitochondrial and cytoplasmic proteins and cytochrome *c* detection

Bos2 cells in a concentration of 1 × 10^6^ in 10 ml of culture medium were seeded in 100-mm cell culture dishes and inoculated at the next day with RML6 or CD1 (0.2%) or treated with 1 μM staurosporine or 10 μM BAY117082 (Calbiochem) for 4 h. Preparations of cytoplasmic and mitochondrial protein extracts were performed and cytochrome *c* was visualized by Western blot analysis as previously described (Liptay *et al*., 2002) using an anti-cytochrome *c* antibody (BD Biosciences) at the final concentration of 1 μg ml^−1^.

### Immunofluorescence

Bos2 and ScN2A cells were cultured on glass coverslips in a 24-well plate and inoculated at the next day with RML6 or CD1 (0.2%) or treated with 1 μM staurosporine or 10 μM BAY117082 for 4 h. Cells were fixed using 4% paraformaldehyde for 20 min at room temperature, washed three times with PBS and permeabilized in −20°C cold methanol on ice for 2 min. Non-specific immunostaining was blocked by incubating the cells in 1% BSA, 10% goat serum in TBS. Active caspases-9 and caspase-3 were detected using antibodies directed against cleaved caspase-9 and cleaved caspase-3 (New England Biolabs) at 1/300 dilution in 1% BSA in TBS and incubated overnight at 4°C. After washing, cells were incubated for 1 h at room temperature with an FITC-conjugated goat anti-rabbit IgG (Dianova) at a 1/3000 dilution in 1% BSA in TBS. After washing the cells were incubated with 0.2 μg ml^−1^ 4′,6′-diamino-2-phenylindole (DAPI; Sigma) for 5 min. The cells were washed, mounted and analysed using an Axiovert microscope and imaging system from Zeiss.

### Western blot analysis of PrP

Brain tissue of PrP^Sc^-infected mice was homogenized in 0.32 M sucrose with a microhomogenizer. For analysis of PrP^Sc^ in terminal sick mice, 1 mg of homogenate in 2% sarkosyl was treated with benzonase (0.1 U/ml, 30 min, 37°C) following proteinase K treatment (20 μg ml^−1^, 30 min, 37°C). Proteins were precipitated using phosphotungic acid (0.28% in 11.8 mM MgCl_2_, 30 min, 37°C). Samples were centrifuged (30 min, 18 000 *g*) and the pellets were resuspended in 20 μl of 0.1% sarkosyl. After dissolution 8 μl of loading buffer (Rotiload, Roth) was added and the extracts were heated at 95°C for 5 min. A total of 250 μg of proteinase K-treated and -precipitated brain homogenate and 50 μg of precipitated but not digested homogenate were subjected to SDS-12.5% polyacrylamide-gel electrophoresis. For comparison at 112 days p.i., protein concentrations were determined by the Bradford method (Bio-Rad) and exact 50 μg of proteinase K-treated brain homogenate (20 μg ml^−1^, 60 min, 37°C) and 50 μg of not digested homogenate were used. Proteins were transferred to Immobilon P membrane (Millipore). Membranes were blocked with Tris-buffered saline containing 0.1% Tween 20 and 5% non-fat milk, incubated with monoclonal antibody POM1 (kindly provided by Dr Aguzzi, Zürich) and detected by ECL (Santa Cruz Biotechnology).

### Microarray analysis

Bos2 cells in a concentration of 1 × 10^6^ in 10 ml of culture medium were seeded in 100-mm cell culture dishes and infected at the next day with RML6 or CD1 (0.2%). RNA was extracted at 2, 8, 18 and 30 h after treatment, using Trizol reagent (Invitrogen) according to manufacturer's instructions. To assure biological relevance pooled RNA was extracted from three independent experiments and was used for analyses. The PIQOR™ Cell Death Microarray (Memorec) was used that is composed of genes encoding key proteins in apoptosis and stress-like caspases, TNF-receptor family members, Bcl-2 family members and heat shock proteins. The microarray was performed and analysed by Memorec.

### Real time qPCR

RNA samples derived from CD1-treated and PrP^Sc^-infected Bos2 cells were used to synthesize single-stranded cDNAs. Reverse transcription was carried out in a reaction volume of 20 μl containing 1 μg of total RNA, 120 ng of random hexamer primer (Amersham Biosciences), 0.5 mM dNTPs (Promega), 10 U RNasin® (Promega), 10 mM DTT, 200 U SuperScript™ II reverse transcriptase (Invitrogen), and the reaction buffer supplied with the enzyme. Negative control reactions were carried out for each sample by replacing the enzyme with water. The mixture was incubated at 25°C for 10 min, 42°C for 50 min and 70°C for 15 min. Real time qPCR was performed using the ABI PRISM® 7000 Sequence Detection System (Applied Biosystems, Darmstadt, Germany). SYBR® Green PCR Master Mix (Applied Biosystems) was used for PCR amplification and real time detection of PCR products. Primers (MWG Biotech) specific for murine *bcl-xl* were designed to have a melting temperature of 60°C (forward primer: 5′-GTCAGCCAGAACCTTATCTTGG-3′, reverse primer: 5′-GCTCAACCAGTCCATTGTCC-3′). 18S RNA was chosen as a reference for normalizations (forward primer: 5′-CGGCTACCACATCCAAGGAA-3′, reverse primer: 5′-GCTGGAATTACCGCGGCT-3′). PCRs were carried out in 20 μl with 300 nM of each primer and with the following temperature profile: 50°C for 2 min, 95°C for 10 min and 40 cycles of 95°C for 15 s and 60°C for 1 min. All samples were amplified in duplicate. Formation of undesired side products during PCR that contribute to fluorescence was excluded by melting curve analysis after PCR. Expression difference between CD1-treated and RML6-infected Bos2 cells for the *bcl-xl* gene was calculated from PCR amplification curves by relative quantification using the comparative threshold cycle (C_T_) method (http://docs.appliedbiosystems.com/pebiodocs/04303859.pdf). The comparative C_T_ method may be used when PCR amplification efficiencies for target and reference primer pairs are similar and close to 1. This was verified by serially diluting cDNA samples, performing qPCR with the different primer pairs and calculating the amplification efficiencies from the slope of the line obtained by plotting C_T_ values versus the logarithm of relative cDNA concentrations.

### Immunohistochemistry

Brain samples were obtained at different time points after infection and fixed in 4% paraformaldehyde (PFA) for 24–72 h, inactivated for 1 h with 98% formic acid, again fixed for 24–72 h with PFA and embedded in paraffin. All tissue sections were stained with haematoxylin-eosin. Immunohistochemistry was carried out as described earlier (Olschlager *et al*., 2004). Briefly, a cleaved caspase-3-specific antibody was used in a 1/200 dilution. A cleaved caspase-9 antibody was used at 1/1000 dilution. A Bcl-x_L_-specific antibody (NEB) was used in a 1/300 dilution and for the detection of activated p65 a phospho-specific antibody [pNF-κB(p65) NEB] at a 1/50 dilution was used. Staining reaction was enhanced by the use of a biotinylated secondary antibody (1/200). For detection an ABC kit was used as described by the manufacturer (Vector). For the detection of active caspase-9 the signal was amplified prior to substrate staining by the use of a TSA indirect kit (Perkin Elmer).

### Immunohistological quantification

To quantify active caspase-3 and Bcl-x_L_ reactivity the total number of labelled cells in three different areas (1.87 mm^2^) of midsagittal sections of three animals were counted by two independent individuals. The three chosen areas, pons/medulla, entorhinal cortex and cerebellum correspond to regions where the vacuolation was found in terminally diseased mice. A total of nine sections were quantified for each mouse strain and the mean value and SEM was calculated and compared with the wild-type C57Bl/6 mice in percentage of control.

## Supplementary material

The following supplementary material is available for this article online:

Fig. S1 Activation of caspase-9 in GT1 cells. (A) Detection of PrPSc in Bos2 and GT1 cells after RML7 inoculation using the cell blot technique as described in Mat & Met. For each experiment the test was performed in duplicate. The circular blots before (left) and after proteinase K treatment (right) of one representative experiment are shown. (B) Detection of active caspase-9 in untreated Bos2, GT1 and ScN2a cells. The active caspase staining is shown in green and the nuclear DAPI staining is shown in blue. Cells presenting chromatin condensation are indicated with yellow arrows. (C) Quantitative analysis of active caspase-9 in Bos2, GT1 and ScN2A cells. Cleaved caspase-9 positive cells were given in % of control (untreated Bos2 cells). The cells from one of three representative experiments/50 mm^2^ were counted.

Fig. S2 Kaplan Meier curves for the onset of disease of wt, NF-κB2- and Bcl-3-deficient mice. Bcl3^-/-^, Nfkb2^-/-^ and C57/Bl6 mice as control were inoculated intracerebrally with 30 μl of 0.2% (w/v) RML6 brain homogenate, monitored daily for clinical symptoms and euthanized at the end point of neurological disease. First disease symptoms were found earlier in NF-κB2- and Bcl-3 deficient mice compared to C57Bl/6 mice.

This material is available as part of the online article from http://www.blackwell-synergy.com/doi/abs/10.1111/j.1462-5822.2007.00950.x
