# Supplementary material for: Alteration of NF-κB activity leads to mitochondrial apoptosis after infection with pathological prion protein
Source: Cell Microbiol. 2007 Jun 15;9(9):2202–17. doi: 10.1111/j.1462-5822.2007.00950.x (PMC2048569; doi:10.1111/j.1462-5822.2007.00950.x)
Supplement: Fig. S1 — Activation of caspase-9 in GT1 cells. (A) Detection of PrPSc in Bos2 and GT1 cells after RML7 inoculation using the cell blot technique as described in Mat & Met. For each experiment the test was performed in duplicate. The circular blots before (left) and after proteinase K treatment (right) of one representative experiment are shown. (B) Detection of active caspase-9 in untreated Bos2, GT1 and ScN2a cells. The active caspase staining is shown in green and the nuclear DAPI staining is shown in blue. Cells presenting chromatin condensation are indicated with yellow arrows. (C) Quantitative analysis of active caspase-9 in Bos2, GT1 and ScN2A cells. Cleaved caspase-9 positive cells were given in % of control (untreated Bos2 cells). The cells from one of three representative experiments/50 mm2 were counted. [file cmi0009-2202-SD1.pdf]

Supplementary 1

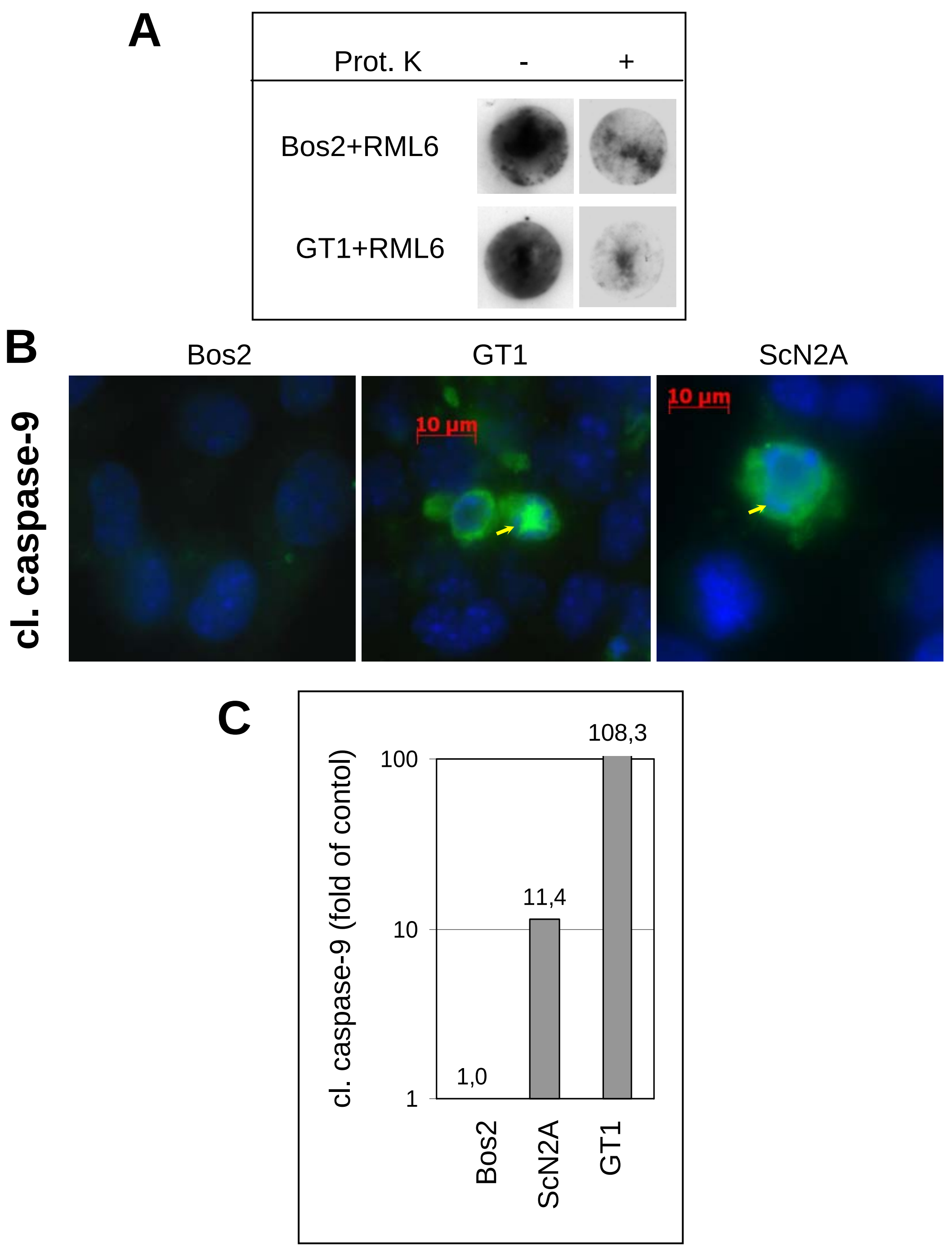

Activation of caspase-9 in GT1 cells. (A) Detection of PrP^{Sc} in Bos2 and GT1 cells after RML7 inoculation using the cell blot technique as described in Mat & Met. For each experiment the test was performed in duplicate. The circular blots before (left) and after proteinase K treatment (right) of one representative experiment are shown. **(B)** Detection of active caspase-9 in untreated Bos2, GT1 and ScN2a cells. The active caspase staining is shown in green and the nuclear DAPI staining is shown in blue. Cells presenting chromatin condensation are indicated with yellow arrows. **(C)** Quantitative analysis of active caspase-9 in Bos2, GT1 and ScN2A cells. Cleaved caspase-9 positive cells were given in % of control (untreated Bos2 cells). The cells from one of three representative experiments/50 mm^2 were counted.

Supplementary 2

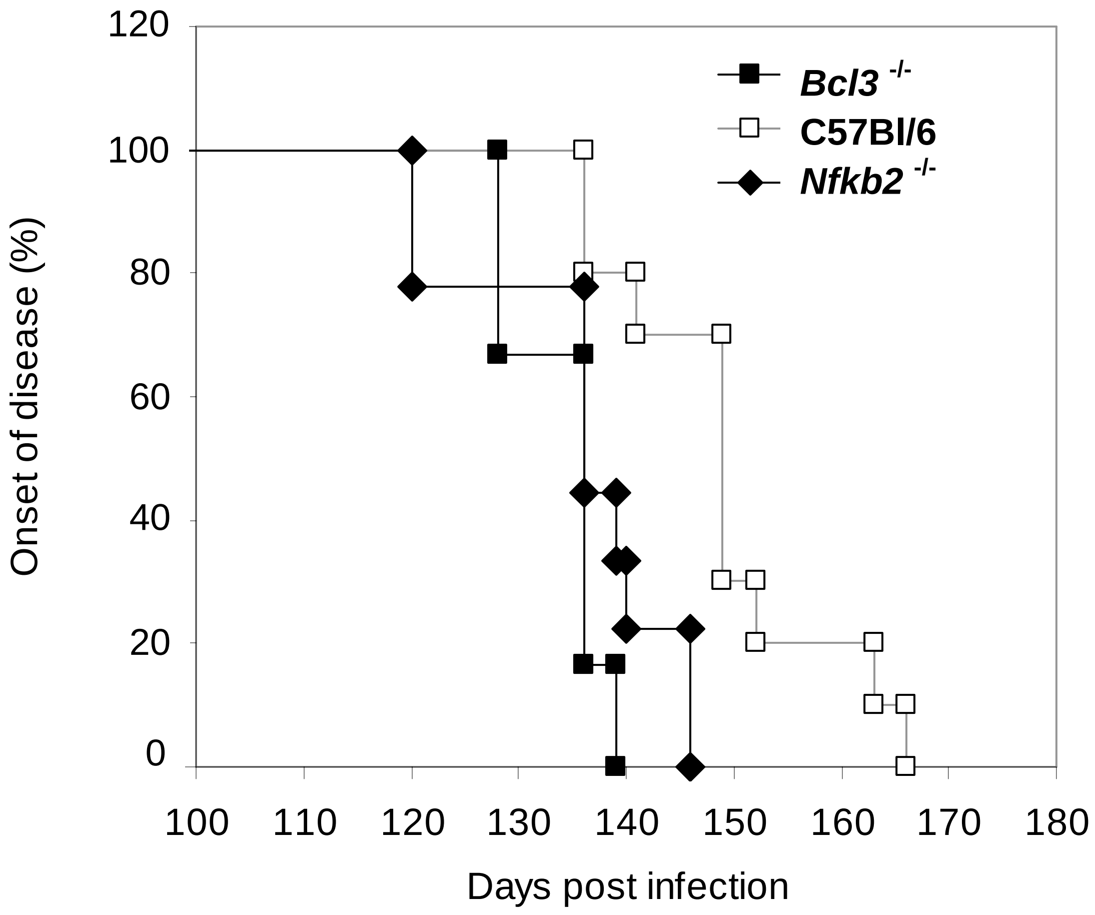

Kaplan Meier curves for the onset of disease of wt, NF-κB2- and Bcl-3- deficient mice. *Bcl3*$^{-/-}$, *Nfkb2*$^{-/-}$ and C57/Bl6 mice as control were inoculated intracerebrally with 30 µl of 0.2% (w/v) RML6 brain homogenate, monitored daily for clinical symptoms and euthanized at the end point of neurological disease. First disease symptoms were found earlier in NF-κB2- and Bcl-3 deficient mice compared to C57Bl/6 mice.
